# Genes Associated with Honey Bee Behavioral Maturation Affect Clock-Dependent and -Independent Aspects of Daily Rhythmic Activity in Fruit Flies

**DOI:** 10.1371/journal.pone.0029157

**Published:** 2012-05-11

**Authors:** Chen Fu, Charles W. Whitfield

**Affiliations:** 1 Neuroscience Program, University of Illinois at Urbana-Champaign, Urbana and Champaign, Illinois, United States of America; 2 Department of Entomology, University of Illinois at Urbana-Champaign, Urbana and Champaign, Illinois, United States of America; AgroParisTech, France

## Abstract

**Background:**

In the honey bee, the age-related and socially regulated transition of workers from in-hive task performance (e.g., caring for young) to foraging (provisioning the hive) is associated with changes in many behaviors including the 24-hour pattern of rhythmic activity. We have previously shown that the hive-bee to forager transition is associated with extensive changes in brain gene expression. In this study, we test the possible function of a subset of these genes in daily rhythmic activity pattern using neural-targeted RNA interference (RNAi) of an orthologous gene set in *Drosophila melanogaster*.

**Principal Findings:**

Of 10 genes tested, knockdown of six affected some aspect of locomotor activity under a 12 h∶12 h light:dark regime (LD). *Inos* affected anticipatory activity preceding lights-off, suggesting a possible clock-dependent function. *BM-40-SPARC, U2af50* and *fax* affected peak activity at dawn without affecting anticipation or overall inactivity (proportion of 15-min intervals without activity), suggesting that these effects may depend on the day-night light cycle. *CAH1* affected overall inactivity. The remaining gene, *abl*, affected peak activity levels but was not clearly time-of-day-specific. No gene tested affected length of period or strength of rhythmicity in constant dark (DD), suggesting that these genes do not act in the core clock.

**Significance:**

Taking advantage of *Drosophila* molecular genetic tools, our study provides an important step in understanding the large set of gene expression changes that occur in the honey bee transition from hive bee to forager. We show that orthologs of many of these genes influence locomotor activity in *Drosophila*, possibly through both clock-dependent and -independent pathways. Our results support the importance of both circadian clock and direct environmental stimuli (apart from entrainment) in shaping the bee’s 24-hour pattern of activity. Our study also outlines a new approach to dissecting complex behavior in a social animal.

## Introduction

Understanding the mechanisms that underlie behavioral maturation in social animals is an important but difficult task. In the honey bee worker, behavioral maturation involves a transition from in-hive task performance to foraging outside the hive [1]. This transition is associated with many behavioral changes, including phototaxis, foraging strategy and daily rhythmic locomotor behavior. Mechanisms that underlie the onset of foraging have been studied intensively. Two circulating factors, juvenile hormone (JH) and the protein vitellogenin [2], [3] act in the onset of foraging and are thought to act as mutual repressors [2], [4]. Foragers have higher titers of JH and lower vitellogenin than hive bees; treatment with juvenile hormone analog or knockdown of vitellogenin by RNA interference accelerate the onset of foraging [5]–[7]. These physiological changes presumably act via the brain to cause changes in an extensive repertoire of behaviors, including transition from an arrhythmic pattern of activity in hive bees to a pattern of activity that is strongly linked to the day-night cycle in foragers [8]. Microarray studies [9]–[11] have identified large sets of gene expression changes in the brain associated with behavioral maturation in the honey bee. However, it is not known which of these genes affect specific behaviors that are part of the foraging repertoire. Here we examine a subset of these genes for possible function in an animal’s 24-hour pattern of locomotor activity.

Rhythmic locomotor activity in a natural day-night setting is likely to result from a complex interplay between clock entrainment, the core endogenous clock (the “pacemaker”), clock output pathways, and so-called “masking” effects (direct environmental effects apart from entrainment of the clock [12]). Studies in *Drosophila* have been instrumental in identifying the core clock genes and genes involved with clock entrainment and output pathways [13]. However, there have been remarkably few genetic studies in *Drosophila* on the role of genes in masking (two examples are [14], [15]).

Many genes involved in these processes are conserved between the honey bee and *Drosophila,* including many of the endogenous clock genes [16]. Studies in the honey bee have focused primarily on changes in the core clock machinery during the switch from arrhythmic to circadian activity pattern [17]–[19]. These studies have shown a link between expression of clock genes and development of circadian rhythmicity in foragers. Further, they have demonstrated that social environment interacts with the clock to affect circadian phenotype [20], [21]. Understanding how daily locomotor patterns develop and change in social species like the honey bee will likely require identification and functional understanding of genes affecting locomotor activity at many different levels, including both clock-dependent and -independent pathways.

A productive approach in studies of honey bee behavioral maturation has been to use gene-behavior information derived in *Drosophila melanogaster* to identify potential genes of importance in honey bee behavior. This approach has been used to identify two honey bee genes (orthologs of *foraging* and *malvolio*) that change expression in the onset of foraging and influence its timing [22], [23]. In the present study, we reverse this strategy by analyzing a set of genes associated with the onset of foraging in the honey bee for possible function using *Drosophila* as a test system. We have previously identified genes from microarray studies of honey bee brains that are good candidates for influencing the onset of foraging or specific foraging related behaviors (see gene selection criteria in Methods). To explore possible function of these genes in daily activity pattern, we tested orthologs of 10 of these genes in *Drosophila* using neural targeted RNA interference (RNAi). These included genes that function in neural development (*abl*, *fax*, *BM-40-SPARC*), neural modulator metabolism (*ple*), other metabolic processes (*CAH1*) or mRNA processing (*U2af50*), and genes with protein similarity or containing protein domains that suggest possible function in second messenger or other signal transduction processes (*Inos*, *Sh3β,* CG32703, CG6910) [24]. Of the genes tested, only *ple* was previously shown to affect locomotor behavior [25]. Our results indicated that a surprisingly large fraction of these genes affect daily rhythmic locomotor activity, likely affecting both endogenous clock-dependent and -independent pathways. These results suggest that a large proportion of gene expression changes in the honey bee brain during behavioral maturation may be associated with modulation of a bee’s 24-hour pattern of locomotor behavior.

## Methods

### Selection of Genes to Test

We used a set of criteria previously described [10] to obtain a list of candidate genes most likely to play a functional role in the onset of foraging in honey bees, based on analyses of brains across several microarray studies. This list includes six genes up-regulated in the transition from hive bee to forager, GB12876, GB11572, GB15888, GB11031, GB14956 and GB15303 corresponding to fly orthologs *U2af50* (*U2 small nuclear riboprotein auxiliary factor 50*), *Inos, CAH1* (*Carbonic anhydrase 1*), CG32703, CG6910 and *ple* (*pale*), respectively, and four genes down-regulated in this transition, GB11301, GB17380, GB19996 and GB11432 corresponding to fly orthologs *abl* (*Abl tyrosine kinase*), *fax* (*failed axon connections*), *Sh3β* and *BM-40-SPARC*. We used three criteria in selecting these genes. First, they were among the most predictive genes for assigning individual bees to behavioral group (hive bee versus forager) [9] and showed consistent expression in an independent microarray study [10]. Second, they were not regulated by flight, light or other foraging-related experience [10]. Third, they were regulated by a juvenile hormone analog in a direction consistent with expression changes (up-regulated for genes higher in forager brains or down-regulated for genes higher in hive bee brains; all genes listed except GB15303) [10]. Orthologs were determined by best match in reciprocal BLASTP searches between *Drosophila melanogaster* and *Apis mellifera* predicted protein sets.

### *Drosophila* Strains and RNA Interference

UAS RNAi responder strains for the genes tested (*abl*, *BM-40-SPARC, CAH1, fax, Inos, U2af* 50, *Sh3β*, *ple,* CG32703 and CG 6910) were ordered from the Vienna Drosophila Research Center (VDRC; Transformant IDs indicated in Table 1; all constructions on *w* background) [26]. The nervous system-specific driver strain *w*; *elav-Gal4* (stock #8760) was backcrossed for five generations with *w*; *TM3, Sb*/*Dr* (kindly provided by Dr. S. A. Kreher). To generate the RNAi genotype for testing, backcrossed *w*; *elav-Gal4* flies (female) were crossed with the respective responder strain to generate heterozygous RNAi flies. For all genes except *abl* and *CAH1*, male flies were tested. For *abl* and *CAH1,* the UAS RNAi responder construct was on the X chromosome and only females could be tested (with the transgene passed from the paternal X). For activity recording, RNAi group and control lines (driver and responder) were tested in parallel for each gene, using flies of the same age and gender. Driver flies were a mixture of homozygous *w*; *elav-Gal4* and heterozygous *w*; *elav-Gal4/TM3, Sb* from the backcross. Responder flies were the original VDRC strains.

**Table 1:** Activity patterns under light-dark (LD) regime.

						Dawn		Dusk		Inactivity (%)
Gene	TID	sex	trials		*n*	Peak	Anticipation	Peak	Anticipation	
***BM-40-***	16678	m	5	*RNAi*	35	**52.9±2.5**	0.78±0.02	65.6±3.7	0.96±0.01	56.3±1.8
***SPARC***				*UAS*	30	**42.8±2.1**	0.73±0.03	58.0±2.7	0.96±0.17	58.5±2.0
				*GAL4*	29	**39.3±2.5**	0.73±0.04	37.9±3.0	0.88±0.02	50.3±2.4
						**	n.s.	n.s.	n.s.	n.s
CG32703	13444	m	2	*RNAi*	11	40.8±3.3	0.82±0.04	49.4±2.0	0.94±0.02	62.5±2.2
				*UAS*	16	48.0±2.6	0.85±0.03	49.5±2.1	0.95±0.02	60.6±3.1
				*GAL4*	11	36.2±4.1	0.80±0.04	38.3±3.2	0.88±0.02	49.1±4.9
						n.s.	n.s.	n.s.	n.s.	n.s.
CG6910	22465	m	3	*RNAi*	14	39.7±2.2	0.72±0.08	45.6±3.1	0.85±0.06	60.3±3.8
				*UAS*	34	36.1±1.6	0.72±0.03	46.8±2.1	0.90±0.02	57.1±2.7
				*GAL4*	34	44.8±2.6	0.60±0.03	37.1±3.7	0.81±0.02	36.7±3.4
						n.s.	n.s.	n.s.	n.s	n.s
***fax***	21895	m	2	*RNAi*	14	**46.6±2.1**	0.86±0.03	57.6±3.0	0.96±0.01	62.2±2.8
				*UAS*	24	**33.5±1.0**	0.75±0.04	59.3±3.0	0.90±0.02	61.9±1.5
				*GAL4*	22	**38.7±1.9**	0.83±0.03	44.2±3.0	0.88±0.02	49.4±1.8
						*	n.s.	n.s.	n.s.	n.s.
***Inos***	5617	m	2	*RNAi*	11	46.4±3.7	0.73±0.05	56.0±5.1	**0.89±0.03**	56.2±4.5
				*UAS*	24	38.6±3.1	0.58±0.02	43.2±3.3	**0.73±0.02**	41.8±2.7
				*GAL4*	22	32.1±2.5	0.68±0.03	34.1±3.1	**0.77±0.03**	41.2±2.8
						n.s.	n.s.	n.s.	**	n.s
*ple*	3308	m	2	*RNAi*	16	38.0±2.7	0.93±0.02	41.5±3.1	0.90±0.03	68.9±1.5
				*UAS*	30	45.6±1.9	0.89±0.01	51.6±2.1	0.96±0.01	61.6±1.9
				*GAL4*	25	36.4±3.3	0.76±0.04	29.6±3.4	0.92±0.02	44.0±3.5
						n.s.	n.s.	n.s.	n.s.	n.s.
*Sh3β*	35970	m	2	*RNAi*	22	50.5±3.3	0.69±0.04	39.2±3.5	0.91±0.03	56.5±3.4
				*UAS*	17	32.7±2.7	0.67±0.03	35.7±3.6	0.75±0.07	48.7±3.7
				*GAL4*	18	42.6±3.9	0.63±0.05	37.4±5.3	0.81±0.03	42.8±4.5
						n.s.	n.s.	n.s.	n.s.	n.s.
***U2af50***	24176	m	3	*RNAi*	14	**51.3±4.3**	0.79±0.04	51.2±4.8	0.95±0.02	55.5±4.1
				*UAS*	39	**39.3±1.5**	0.79±0.02	45.7±2.7	0.93±0.02	60.6±2.0
				*GAL4*	39	**36.3±2.0**	0.67±0.04	34.8±2.9	0.87±0.02	45.3±3.1
						**	n.s.	n.s.	n.s.	n.s.
***abl***	2897	f	2	*RNAi*	36	**43.4±1.6**	0.77±0.02	**40.4±2.0**	0.74±0.03	33.8±2.3
				*UAS*	33	**32.1±2.1**	0.67±0.05	**23.9±1.9**	0.59±0.02	53.1±2.1
				*GAL4*	33	**31.6±1.5**	0.70±0.03	**28.9±2.1**	0.68±0.02	37.4±2.6
						**	n.s.	**	n.s.	n.s.
***CAH1***	26015	f	2	*RNAi*	28	36.5±2.5	0.81±0.03	32.1±1.9	0.68±0.02	**28.8±2.4**
				*UAS*	31	36.3±1.6	0.79±0.02	29.1±2.2	0.57±0.02	**39.4±1.8**
				*GAL4*	30	30.6±1.7	0.73±0.05	30.1±1.7	0.78±0.02	**39.3±2.0**
						n.s.	n.s.	n.s.	n.s.	*

Significance is indicated at the gene-level threshold, *p*<0.005 (*), or the experiment wide threshold, *p*<0.0005 (**). For all effects reported as significant, RNAi group differed from both control groups in the same direction (*p*<0.05; *post hoc*). Significant effects are highlighted by bold text. Peak activity, anticipation and inactivity are defined in Methods. Number of individual flies (*n*) is indicated for the experimental F_1_ RNAi flies and the two control lines (*elav-Gal4* driver and the gene-specific *UAS* responder line). TID, Transformant ID; n.s., not significant.

Efficiency of RNAi was measured by real-time quantitative reverse transcription PCR of single whole *Drosophila* heads using *rp49* as the control gene. cDNA was generated and quantified using ABI-SDS 7900 system as in [27]. PCR reactions contained 3 µl targeted cDNA (10–100 ng), 5 µl Syber-green mix and 2 µl primer pair (2.5 µM). 3 µl of each reaction was added to 2 or 3 wells in the 384-well reaction plate. mRNA reduction was calculated by 1–2^−ΔΔCt^, where ΔΔC_t_ = ΔC_t,RNAi_–ΔC_t,control_ and ΔC_t_ was the difference in mean threshold cycles between target gene and *rp49*.

Efficiency was tested for a total of seven genes: *U2af50, Inos, CAH1, fax, BM-40-SPARC, abl* and CG32703 (using primers indicated in Table S1). RNAi and control flies were collected in parallel for mRNA quantification either as siblings of the behaviorally tested flies (collected at 1 or 2 days of age) or were the behaviorally tested flies collected immediately after the DD regime. Control group for mRNA quantification was driver or responder strain (see Table S1) of the same age and gender as the RNAi group. Reduction of mRNA in single whole heads varied from 35% to 90% and was significant for all seven genes tested (*p<*0.05; Table S1).

### *Drosophila* Activity Recording

One- or 2-day-old flies from the RNAi group and control groups (driver and responder lines) were put into the *Drosophila* Activity Monitor (Trikenetics, Inc). Locomotor activity was recorded by computer as in [28]. The flies experienced 2 days light:dark (LD) entrainment (12 h∶12 h) and activity was recorded over the subsequent 5 days of LD. Flies were then shifted to a constant dark regime (DD) and activity recorded for 5 more days.

### Measurement of Behavioral Parameters

A total of five behavioral parameters were derived from individual fly activity in LD condition and five additional parameters in DD. In all cases, parameters were estimated for single flies (after exclusion of dead flies) using activity data over 5 days of LD or 5 days of DD; individual values were then used in statistical analyses below. Several parameters were calculated from unsmoothed activity data including inactivity (proportion of 15-min intervals with no activity) for both LD and DD and anticipation indices for lights-on and lights-off (LD only). Anticipation index was calculated as in [29] by dividing the sum of activity during 3 hours preceding light change by the sum of activity during 6 hours preceding light change.

Peak activity at dawn and dusk (LD) and subjective dawn and dusk (DD) were calculated from smoothed activity data for each fly. Activity was smoothed using a non-recursive linear digital low-pass filter that has been used in *Drosophila* activity studies and is not expected to cause phase shift [30]. Smoothed activity at each 15-min time interval (*Y_i_*) was calculated using the formula *Y_i_ = X_i_+fc_1_ (X_i+1_+X_i−1_)+fc_2_ (X_i+2_+X_i−2_)+fc_3_ (X_i+3_+X_i−3_),* (i = 4 to 477 in this study) where *fc_j_ = sin(2πj/rt_c_)/(2πj/rt_c_), j* = 1 to 3, *r = *4 h^−1^(sampling rate per hour) and *t_c_ = *2 h (cut-off period). This formula was applied using R.2.90. Peak activity at each dawn or dusk period was determined as the highest activity in the smoothed plot in the 5.5 h period centered on Zeitgeber time 0 (dawn) or 12 (dusk). Peak dawn and dusk activities were then calculated for each fly as the average over the 5 day recording period.

Strength of rhythmicity (amplitude) and length of period (*tau*) for each fly were estimated from DD activity using the LSP program [31]. Only flies with χ^2^ periodogram (Qp) significant at *p*<0.01 were used in statistical analyses of *tau* and amplitude.

### Statistical Analysis

Proportion of intervals with no activity and anticipation indices were arc-sine transformed. ANOVA was performed using R.2.90 package to test for differences between the three groups tested (RNAi, driver and responder) treating trial and group as factors. To address multiple testing, we used Bonferroni adjustments to calculate two critical significance thresholds. The first threshold, referred to as gene-level significance, accounted for the 10 behavioral parameters tested for each gene (α = 0.05; *p*<0.005). We consider this a marginal significance level. The second threshold, referred to as experiment-wide significance, accounted for the 10 parameters and the 10 genes examined (α = 0.05; *p*<0.0005). Results that were significant at either threshold were examined *post hoc* to ensure that RNAi group differed significantly (*p*<0.05) from both control groups in the same direction; only results meeting this standard are reported as significant.

## Results

**Figure 1:**
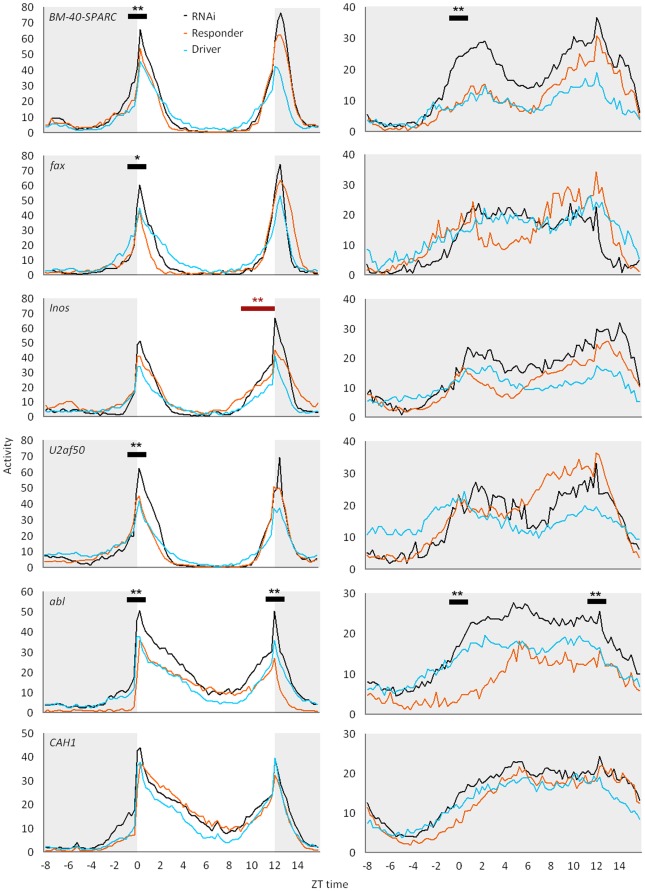
Activity patterns in light-dark (LD) and constant dark following entrainment (DD). Plots show unsmoothed activity averaged across individual flies and the 5-day recording period under LD (left panels) and DD (right panels). Shading indicates dark period. RNAi, Responder and Driver genotypes are described in Methods. All genes were tested in males except for *Abl* and *CAH1,* which were tested in females. Significant effects on peak activity are indicated by black bar (asterisks as in Table 1 and 2). Significant effect on anticipatory activity is indicated by a red bar. Additional effects on inactivity for *CAH1* (in LD) and *BM-40-SPARC* (in DD) are not indicated in figure (see Table 1 and 2).

We tested neural-targeted RNAi lines for the selected 10 genes (see Methods) for effects on different aspects of locomotion in LD (Table 1) and DD following LD entrainment (Table 2 and 3). A total of six genes affected some aspect of locomotion in LD, significant at the gene-level or experiment-wide thresholds, *p<*0.005 or 0.0005, respectively (ANOVA; *post hoc* showed RNAi group differed from both control groups, *p*<0.05). Two of these genes also affected locomotion in DD. RNAi and control lines for these six genes are shown in Fig. 1, with activity averaged across replicate flies and over the 5 day LD period (left panels) or 5 day DD period (right panels).

**Table 2:** Activity patterns under constant dark (DD) regime.

						Peak activity		
Gene	TID	sex	trials		*n*	Subj. dawn	Subj. dusk	Inactivity (%)
***BM-40-***	16678	m	4	*RNAi*	16*	**45.9±3.1**	34.0±2.2	**23.5±2.2**
***SPARC***				*UAS*	20	**32.7±2.2**	28.3±2.3	**42.3±2.5**
				*GAL4*	16	**19.6±2.2**	19.3±1.9	**36.0±4.8**
						**	n.s.	**
CG32703	13444	m	2	*RNAi*	10	32.1±2.6	38.6±2.7	31.5±2.3
				*UAS*	15	37.9±4.3	38.5±3.0	37.0±3.4
				*GAL4*	7	19.3±3.4	21.7±1.8	25.8±5.5
						n.s.	n.s.	n.s.
CG6910	22465	m	1	*RNAi*	8	24.2±3.1	35.0±3.6	50.0±2.3
				*UAS*	16	15.8±1.8	32.7±1.9	58.8±3.1
				*GAL4*	15	25.6±4.0	30.4±4.4	23.5±4.4
						n.s.	n.s.	n.s.
*fax*	21895	m	2	*RNAi*	8	33.4±3.0	37.5±3.4	38.7±2.2
				*UAS*	16	22.4±3.2	42.9±2.4	46.0±3.4
				*GAL4*	17	27.3±2.1	34.7±4.1	28.1±3.1
						n.s.	n.s.	n.s.
*Inos*	5617	m	2	*RNAi*	5	35.0±5.8	38.8±3.9	35.2±5.7
				*UAS*	12	23.1±3.1	24.8±3.2	31.0±3.9
				*GAL4*	6	20.6±3.2	19.8±2.3	37.4±9.4
						n.s.	n.s.	n.s.
*ple*	3308	m	1	*RNAi*	8	29.0±5.2	36.3±3.2	41.8±2.9
				*UAS*	16	35.9±2.0	46.8±2.2	35.5±2.3
				*GAL4*	13	28.2±3.0	25.5±3.3	23.9±3.6
						n.s.	n.s.	n.s.
*Sh3β*	35970	m	1	*RNAi*	16	31.2±3.9	31.2±2.9	37.5±4.1
				*UAS*	6	23.8±4.8	31.9±5.3	35.6±6.1
				*GAL4*	8	30.2±3.9	31.3±5.3	12.5±3.2
						n.s.	n.s.	n.s.
*U2af50*	24176	m	2	*RNAi*	9	34.3±3.6	36.8±3.6	28.2±4.4
				*UAS*	20	31.1±2.6	40.0±2.4	37.0±3.5
				*GAL4*	20	29.0±3.1	28.3±3.3	22.2±4.6
						n.s.	n.s.	n.s.
***abl***	2897	f	2	*RNAi*	28	**33.6±1.8**	**32.5±2.0**	18.5±3.5
				*UAS*	19	**21.8±2.9**	**20.9±2.5**	50.0±4.6
				*GAL4*	25	**23.1±2.0**	**23.9±1.8**	25.2±4.0
						**	**	n.s.
*CAH1*	26015	f	2	*RNAi*	11	39.1±3.1	30.8±3.6	20.2±5.2
				*UAS*	16	28.2±3.2	24.2±3.3	22.4±4.8
				*GAL4*	15	31.1±3.3	29.2±2.8	26.2±4.6
						n.s.	n.s.	n.s.

See notes for Table 1. Subjective dawn and dusk are described in Methods.

**Table 3:** Rhythmicity and length of period under DD.

Gene	TID	sex	trials		flies tested	% rhythmic flies	*tau* (hrs)	amplitude (Qp)
*BM-40-*	16678	m	4	*RNAi*	17	100%	24.4±0.5	92.2±8.6
*SPARC*				*UAS*	23	91.3%	24.6±0.1	111.4±7.7
				*GAL4*	19	89.5%	24.8±0.3	77.8±8.2
							n.s.	n.s.
CG32703	13444	m	2	*RNAi*	11	90.9%	25.4±0.6	74.4±5.8
				*UAS*	16	100%	24.5±0.4	90.2±5.8
				*GAL4*	7	85.7%	24.6±0.2	67.2±10.6
							n.s.	n.s.
CG6910	22465	m	1	*RNAi*	8	100%	24.8±0.1	92.2±5.3
				*UAS*	23	100%	24.7±0.1	130.6±6.7
				*GAL4*	23	87.0%	24.9±0.4	69.6±6.2
							n.s.	n.s.
*fax*	21895	m	2	*RNAi*	8	100%	25.2±0.9	74.4±13.0
				*UAS*	16	93.8%	24.9±0.3	98.9±8.6
				*GAL4*	11	90.9%	25.1±0.3	73.4±7.7
							n.s.	n.s.
*Inos*	5617	m	1	*RNAi*	6	100%	25.0±0.2	122.9±21.1
				*UAS*	9	100%	24.2±0.3	97.9±12.5
				*GAL4*	4	100%	25.3±0.3	97.9±16.3
							n.s.	n.s.
*ple*	3308	m	1	*RNAi*	8	100%	25.5±0.1	100.8±8.2
				*UAS*	16	100%	25.2±0.1	106.6±6.2
				*GAL4*	13	92.3%	24.8±0.5	98.9±10.1
							n.s.	n.s.
*Sh3β*	35970	m	1	*RNAi*	16	93.8%	24.4±0.2	101.8±12.5
				*UAS*	6	100%	24.4±0.2	113.3±10.1
				*GAL4*	8	75%	24.0±0.8	38.4±4.3
							n.s.	n.s.
*U2af50*	24176	m	2	*RNAi*	16	93.8%	24.6±0.1	137.3±11.5
				*UAS*	20	95.0%	24.5±0.1	155.5±10.6
				*GAL4*	19	73.6%	24.5±0.5	47.0±4.8
							n.s.	n.s.
*abl*	2897	f	2	*RNAi*	36	97.2%	24.2±0.2	156.5±9.1
				*UAS*	20	85.0%	24.7±0.1	148.8±13.9
				*GAL4*	28	85.7%	23.8±0.1	124.8±11.5
							n.s.	n.s.
*CAH1*	26015	f	2	RNAi	19	95.7%	24.5±0.2	157.4±12.5
				UAS	27	100%	24.7±0.1	156.5±7.7
				GAL4	17	100%	24.6±0.2	156.5±10.1
							n.s.	n.s.

Percent rhythmic flies indicates the proportion of flies with significant rhythmicity (*p*<0.01). Only rhythmic flies were used in statistical analyses of *tau* and amplitude.

Knockdown of *BM-40-SPARC, fax* and *U2af50* increased peak locomotor behavior at dawn in LD (*p<*0.0005), but did not decrease overall inactivity (proportion of 15-min intervals with no activity, *p*>0.005) (Table 1 and Fig. 1). The latter result suggests that increased activity in these lines was not a general increase at all times of the day. Although they did not show significant effects on dusk, two of these genes showed trends in dusk activity (non-significant elevation) that make it difficult to interpret a specific effect on dawn versus dusk activity. Knockdown of *abl* caused increased peak activity at both dawn and dusk (*p<*0.0005); however, control line differences in inactivity make it difficult to rule out a general increase in activity at all times.

Two other genes showed effects under LD conditions. Knockdown of *Inos* caused a significant increase in lights-off anticipatory locomotion (*p*<0.0005; Table 1 and Fig. 1). Knockdown of CAH1 caused a decrease in inactivity at the marginal gene level threshold (*p*<0.005).

Locomotor activity under constant dark (DD) following LD entrainment was affected for two genes (Table 2). RNAi knockdown of *BM-40-SPARC* caused increased activity at subjective dawn (*p*<0.0005) but also a decrease in total inactive time, suggesting that constant dark may have a general activating effect on *BM-40-SPARC* knockdown flies. Knockdown of *abl* increased in peak activities in DD (*p*<0.0005) similar to its effect in LD; however, control line differences in inactivity make it difficult to rule out a general increase in activity.

No gene tested showed differences in strength of rhythmicity or length of period (*tau*) in DD (*p>*0.005; Table 3).

## Discussion

In this study, we tested orthologs of 10 genes associated with honey bee behavioral maturation, finding six that affected some aspect of *Drosophila* locomotor activity. One gene, *Inos*, affected anticipation of lights-off. Three genes, *BM-40-SPARC, fax* and *U2af50*, affected dawn activity without affecting total time spent inactive. Knockdown of *abl* caused increased peak activities, but our data did not strongly support a time-specific effect. Knockdown of *CAH1* caused a marginally significant (gene-level threshold) decrease in time inactive. No genes affected strength of rythmicity or length of period in DD.

These six genes could influence activity via either clock-dependent or direct stimulus-dependent (apart from entrainment; i.e., masking) pathways. Our data suggest that at least one, *Inos*, acts downstream of the endogenous clock. Knockdown of *Inos* affected activity in the 3-hours prior to lights-off (anticipation of dusk), but did not affect strength of rhythmicity or length of period in DD, suggesting a clock-dependent rather than a core clock function [32]. Consistent with a possible role downstream of the clock, *Inos* was identified as significant clock controlled genes in a meta-analysis of *Drosophila* circadian microarray studies [33] (of the 10 genes examined in the present study, *CAH1* and *ple* were also identified as clock controlled genes). Both *BM-40-SPARC* and *abl* showed effects in DD resembling their effects in LD. However, we cannot make a strong interpretation of clock-dependent effect for either of these genes: *BM-40-SPARC* exhibited a general increase in activity in DD, while control line effects in *abl* make it difficult to interpret inactivity. No other gene in this study affected strength of rhythmicity or length of period in DD, suggesting no role in the core clock machinery for genes examined in this study.

Results for the two remaining genes that affected dawn peak activity, *fax* and *U2af50*, were consistent with possible modulation by direct light stimulus rather than the endogenous clock. RNAi of both genes increased activity at dawn without decreasing total time spent inactive, indicating time-specific effects under LD. However, neither gene showed effects on dawn or dusk anticipation, nor activity under DD. These results suggest that observed increases in dawn activity may result from light transition. Results were similar for *BM-40-SPARC*, although unlike *fax* and *U2af50, BM-40-SPARC* may have shown a generalized response (elevated locomotor activity) to DD. Taken together, our results suggest that *fax, U2af50* and *BM-40-SPARC* may mediate direct stimulus effects on activity, though further behavioral tests are needed to establish light masking effects [12].

Considered together, a surprisingly large fraction of genes tested showed effects on the 24-hour pattern of locomotor activity in *Drosophila* (six out of ten), although only one exhibited a clear clock-dependent function. This result may point to the importance (and complexity) of changes in locomotor behavior in the honey bee transition to foraging. The onset of foraging involves both increased overall activity (foraging flight) but also long inactive periods linked to the circadian clock [8]. Perhaps resulting from this complexity, we did not observe a simple correspondence between direction of effect in *Drosophila* (more or less activity) and direction of regulation in the hive bee to forager transition (up or down regulation). An important caveat is that our results do not address how many genes in the *Drosophila* genome would show similar effects. Because genes can act pleiotropically, it is possible that our results reflect a general trend in which a large fraction of genes in the genome have small but measurable effect on some aspect of locomotion in addition to affecting other phenotypes. More detailed understanding of the function of each of these genes in *Drosophila* locomotion may provide insight into their possible specific roles in the complex honey bee foraging phenotype. A full understanding of the 24-hour pattern of locomotor behavior in the honey bee will require an understanding of the genes that act in the endogenous clock, genes that translate the endogenous clock information to locomotor activity, and genes that translate environmental and social cues to locomotor activity (both via clock entrainment and clock-independent pathways).

Although the current study focuses on daily locomotor activity, the general approach could be used to study other behaviors associated with the transition from hive bee to forager, for example in foraging strategy, phototaxis and aggression. Such studies could identify pleiotropic effects of genes implicated in the present study (in locomotor activity) and lead to a deeper understanding of both the mechanism of social behavior and the hierarchy of complex behaviors.

## Supporting Information

Table S1 **2^−ΔΔCt^ represents mRNA abundance in the RNAi group relative to the control group.** * Flies from the responder strain were used as control group. ** Flies from the driver strain were used as control group.
(DOC)
