# Supplementary material for: Genes Associated with Honey Bee Behavioral Maturation Affect Clock-Dependent and -Independent Aspects of Daily Rhythmic Activity in Fruit Flies
Source: PLoS One. 2012 May 11;7(5):e29157. doi: 10.1371/journal.pone.0029157 (PMC3350530; doi:10.1371/journal.pone.0029157)
Supplement: Table S1 — 2−ΔΔCt represents mRNA abundance in the RNAi group relative to the control group. * Flies from the responder strain were used as control group. ** Flies from the driver strain were used as control group. (DOC) [file pone.0029157.s001.doc]

Supplementary Table 1. RNA interference measured by real-time quantitative reverse transcription PCR.

| Gene | Forward primer/Reverse primer | n (RNAi) | n (Control) | 2^-ΔΔCt^ | ΔC_t,RNAi_ | ΔC_t,control_ | *p* |
| --- | --- | --- | --- | --- | --- | --- | --- |
| *BM-40-SPARC* | 5'-CGAAGCTTTTCCACAGGCAA-3'/  5'-CATCTCGAATGGCGATCTCAAT-3' | 12 | 12* | 0.593 | 4.80 | 4.05 | 0.0070 |
| CG32703 | 5'-CCTCCTCCGATACGCATCTACA-3'/  5'-ATGTTTCACTCGGAGACCACG-3' | 12 | 9* | 0.300 | 4.77 | 3.03 | 0.0051 |
| *fax* | 5-TCCAGTTGGCCTTCCGTTTAT-3'/  5'-CTGCGGCTGATGATGATTCTTT-3' | 18 | 17* | 0.671 | 3.05 | 2.47 | 0.018 |
| *Inos* | 5'-TTTTCATTGCCGGCGATG-3'/  5'-TGTAACTGGCAATGGACACCG-3' | 10 | 8* | 0.101 | 9.66 | 6.35 | 0.039 |
| *U2af50* | 5'-TGGCCTAAATGGAATGCAGC-3'/  5'-ATTACGGACTGCGTGGTGTTG-3' | 12 | 12** | 0.541 | 5.00 | 4.11 | 3.7E-06 |
| *abl* | 5'-TCATTAAGCGCAAGTGATCGG-3'/  5'-GCTATCTTTGCTTCCATTTCCG-3' | 12 | 12** | 0.387 | 5.00 | 3.63 | 0.00042 |
| *CAH1* | 5'-GCTGCATTTGGTTCACTGGAA-3'/  5'-CATGGTGATTGCCAGCCTTAA-3' | 12 | 12** | 0.592 | 3.73 | 2.97 | 0.0017 |
| *rp49* | 5'-CCCACCGGATTCAAGAAGTTC-3'/  5'-GCATGAGCAGGACCTCCAG-3' |  |  |  |  |  |  |

2^-ΔΔCt^ represents mRNA abundance in the RNAi group relative to the control group.

* Flies from the responder strain were used as control group.

** Flies from the driver strain were used as control group.
